# A unified platform enabling biomarker ranking and validation for 1562 drugs using transcriptomic data of 1250 cancer cell lines

**DOI:** 10.1016/j.csbj.2022.06.007

**Published:** 2022-06-06

**Authors:** János Tibor Fekete, Balázs Győrffy

**Affiliations:** a Semmelweis University, Department of Bioinformatics and 2^nd^ Department of Pediatrics, Budapest H-1094, Hungary; b Research Center for Natural Sciences, Institute of Enzymology, Momentum Cancer Biomarker Research Group, Magyar tudósok körútja 2., Budapest H-1117, Hungary

**Keywords:** Chemotherapy, Proliferation, In vitro, Receiver operator characteristics, RNAseq, Machine learning, Random forest

## Abstract

**Intro:**

In vitro cell line models provide a valuable resource to investigate compounds useful in the systemic chemotherapy of cancer. However, the due to the dispersal of the data into several different databases, the utilization of these resources is limited. Here, our aim was to establish a platform enabling the validation of chemoresistance-associated genes and the ranking of available cell line models.

**Methods:**

We processed four independent databases, DepMap, GDSC1, GDSC2, and CTRP. The gene expression data was quantile normalized and HUGO gene names were assigned to have unambiguous identification of the genes. Resistance values were exported for all agents. The correlation between gene expression and therapy resistance is computed using ROC test.

**Results:**

We combined four datasets with chemosensitivity data of 1562 agents and transcriptome-level gene expression of 1250 cancer cell lines. We have set up an online tool utilizing this database to correlate available cell line sensitivity data and treatment response in a uniform analysis pipeline (www.rocplot.com/cells). We employed the established pipeline to by rank genes related to resistance against afatinib and lapatinib, two inhibitors of the tyrosine-kinase domain of ERBB2.

**Discussion:**

The computational tool is useful 1) to correlate gene expression with resistance, 2) to identify and rank resistant and sensitive cell lines, and 3) to rank resistance associated genes, cancer hallmarks, and gene ontology pathways. The platform will be an invaluable support to speed up cancer research by validating gene-resistance correlations and by selecting the best cell line models for new experiments.

## Graphical abstract

**Figure:**
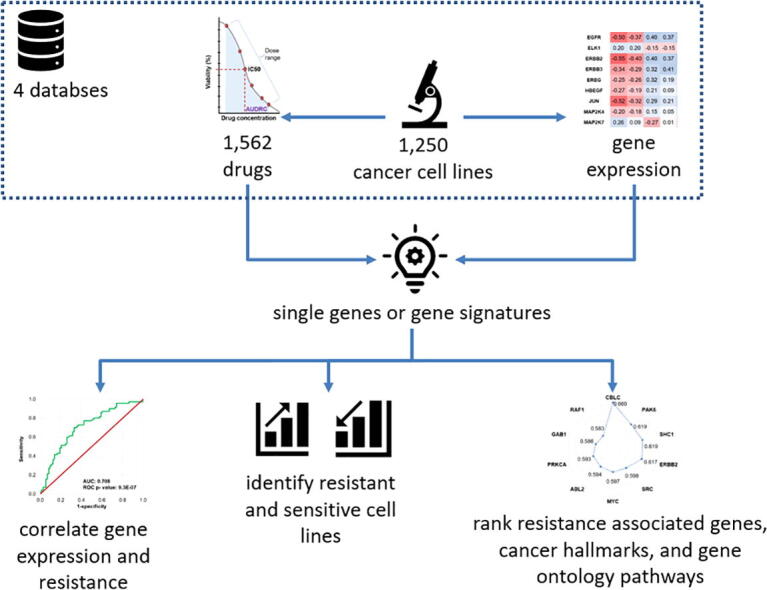

## 1 Introduction

Cancer is considered the second most common cause of death worldwide with 10 million cancer-related deaths and 19.3 million new cancer diagnoses according to an estimation in 2021 [1]. Despite improvements of progression-free and overall survival in the past few years in the treatment of certain tumors, we still see a significant rate of tumor types in which no improvement has been made [2]. The majority of anticancer agents are not universally effective, and they have anti-tumor activities only in distinct groups of tumors. Besides, the therapeutic response varies from person to person, which is determined by factors of genetic and environmental variations. A robust resource enabling the application of personalized therapies is the determination of gene expressions levels that are capable of acting as biomarkers to select patients who will most likely benefit from a given therapy as has been demonstrated for breast cancer endocrine therapy [3].

New drug development can be challenging due to high costs and the fact that the mean time from the initial screening to final approval of the drug can take more than ten years. Approval can be based on drug repurposing as well – in this case a therapy already approved for some indication is assessed for another indication [4]. As these drugs already have a regulatory approval, data on their safety profiles and potential interactions with other drugs are readily available, thus the time and the cost needed to introduce the therapy with a new indication can be significantly reduced. There are several drug repurposing candidates with anticancer potential today. For example, the effects of cardiovascular drugs including aspirin, ACE inhibitors, and beta blockers are now under investigation in oncology [5]. Anticancer indications have also been suggested for psychiatric drugs including valproic acid, phenothiazines, selective serotonin reuptake inhibitors, tricyclic antidepressants, and MAO inhibitors [6]. A rational first step in the discovery and validation of such agents is the preclinical analysis of their effect in cancer cell lines.

Cancer cell lines provide clinically useful data by enabling the experimental investigation and modelling of new treatments and therapy resistance related factors [7]. In the past decades, several cell line databases have been established that enable the linking of pharmaceutical agents to tumor growth inhibition. Initial studies had panels of cell lines with sensitivity data for a handful of agents [8]. Large scale anti-tumor drug screening with more than 21,000 agents tested in sixty cell lines was launched by the National Cancer Institute in the 1990′s [9]. The Cancer Cell Line Encyclopedia (CCLE) project, a cooperation between the Broad Institute and Novartis, provides the genetic and pharmacological characteristics of more than 1100 cell lines [10]. The Cancer Therapeutics Response Portal (CTRP) enables access to 860 cell lines [11] while the Genomics of Drug Sensitivity in Cancer (GDSC) project contains data for more than 1000 cell lines and their interactions with more than 500 drugs [12]. The Cancer Dependency Map (Depmap), a multi-institutional project to map genetic dependencies, provides genetic mapping for more than five hundred cell lines and resistance data for more than 4,000 agents [13].

In our work we have set three goals. First, we integrated data from multiple large-scale cell line databases to establish an easy to use online platform that provides swift access and analysis of the data in order to uncover relationships between gene expression and therapeutic response across a large panel of drugs. Second, we established a ranking of cell lines enabling the identification of the most robust preclinical model. Third, we validated our approach by selecting lapatinib and afatinib, two ERBB2 tyrosine kinase domain inhibitors, which were evaluated in each included dataset, and by ranking the significant gene expression-based biomarkers.

## 2 Methods

### 2.1 Drug screening and gene expression data

For the setup of the database, we collected data from four publicly available drug screening databases. Drug sensitization data of the Cancer Dependency Map Consortium's DepMap portal (https://depmap.org/) were obtained from the PRISM Repurposing 19Q4 secondary screen dose–response dataset [13]. From the Genomics of Drug Sensitivity in Cancer (GDSC) project [14] both GDSC1 and GDSC2 drug screening datasets were taken, whereas from the Cancer Therapeutics Response Portal (CTRP) the version 2 drug screening dataset was obtained [15].

DepMap and CTRP drug screening datasets are based on the CCLE cell lines and for gene expression data the 21Q1 RNAseq data was used as a source [16]. Read count data were normalized with the DESeq algorithm, then a quantile and a scaling normalization method were applied to set the mean gene expression in each cell line to 1000. Genes with a zero-expression value in more than half of the cell lines were excluded from the analysis. For the gene expression of the cell lines in the GDSC drug screening datasets we obtained RMA normalized Affymetrix HGU-219 microarray expression matrix, and applied a second scaling normalization method as above. Pre-processed data were imported into a PostgreSQL database. For the identification of unambiguous gene names, we used the HUGO Gene Nomenclature Committee (HGNC) database (https://www.genenames.org/).

### 2.2 Treatment response categorization

**Fig. 1:**
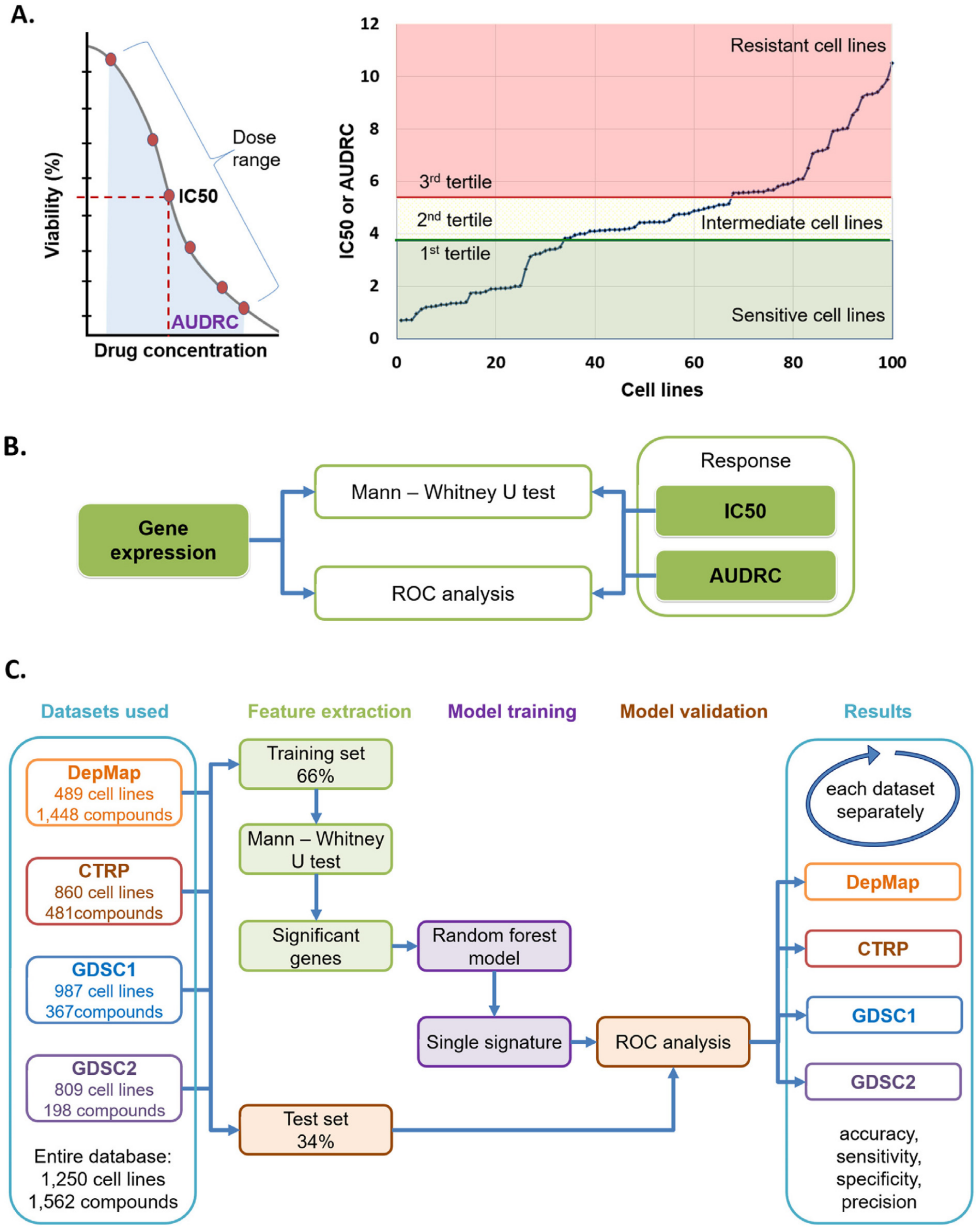
**Overview of the analysis pipeline.** Summary of response classification using IC50 and AUDRC (area under the dose response curve) values (A). Primary statistical methods for single gene analyses (B), and the setup for the machine learning pipeline for gene signature analysis (C).

In the analysis of DepMap and GDSC based projects we used the reported half-maximal inhibitory concentration (IC50) and the area under the dose-response curve (AUDRC) values to evaluate therapeutic response. AUDRC is determined using the dose range spanning from the lowest to the highest applied dose for the drug under investigation. Cases where neither IC50 nor AUDRC was determined were excluded from the analysis. For each agent, we defined lower and upper tertile cutoff values based on the IC50 or AUDRC values and cell lines with IC50 or AUDRC values in the lower tertile were considered as sensitive and those in the upper tertile were considered as resistant. Cells belonging to the intermediate tertile were not considered in the analysis. In the analysis of the CTRP project only AUDRC values were reported and we used it to assess therapeutic response with the median and tertile based method as described above. The difference between IC50 and AUDRC is summarized in Fig. 1A.

### 2.3 General statistical methods

The analysis was performed in the R statistical software environment (https://www.r-project.org/). Mann-Whitney *U* test and receiver operating characteristics (ROC) were computed in order to compare single gene expression values between sensitive and resistant samples (Fig. 1B). Spearman rank correlation was applied to compare published AUDRC values with gene expression. The statistical significance cutoff was set at p < 0.05.

### 2.4 Gene signature analysis

To assess the relation of different pathways and cancer hallmark genes to therapeutic response, we utilized the lists of KEGG pathways (https://www.genome.jp/kegg/) and a previously assembled lists of cancer hallmark genes [17]. In these, 712 genes belonging to seven hallmarks and 4,602 genes belonging to 186 KEGG pathways can be tested per therapeutic agent. Using these genes, the analysis pipeline was extended with a machine learning computation method to analyze the entire signature. As a first step, samples are randomly divided into a training (66%) and a test set (34%). Second, the system selects genes significantly (p < 0.05) correlated to resistance using a Mann-Whitney test. Then, the significant genes are integrated by a random forest classifier into a single signature. Finally, a ROC analysis is used to evaluate the predictive effectiveness of this signature. As a result, the list of genes significant in the signature, the confusion matrix of the test set, and the overall predictive power of the signature including the computed accuracy=(TP + TN)/(TP + FN + FP + FN), sensitivity = TP/(TP + FN), specificity = TN/(TN + FP), and precision = TP/(TP + FP) values are provided (Fig. 1C).

### 2.5 Online analysis portal

We extended our previously established ROC plotter tool [18] with the cell line database. The portal is set up to require the investigated agent and the biomarker candidate as input. Datasets with available treatment and expression data as well as the most robust response data are automatically selected. Using these input parameters, the ROC AUC plot is generated for each available setting. Furthermore, the sensitivity across all available cell lines is provided as a table. In addition to single genes, simultaneous analysis of multiple genes can be performed by using the mean expression of the included genes as described above. When analyzing a set of genes, false discovery rate is computed and provided in the results page.

### 2.6 Sample application: Validation of genes related to sensitivity against lapatinib and afatinib

To validate the robustness of the established database, we aimed to analyze two selected pharmaceutical agents acting on an established therapeutic target. For this validation, we selected the drugs afatinib and lapatinib, both targeting the ERBB2 receptor according the DrugBank database [19]. This selection was based on the fact that sensitivity data for these two agents was available in each of the four included databases. The analysis was performed using the tertile- based therapeutic response categorization as described above.

## 3 Results

### 3.1 Therapeutic agents in the database

**Fig. 2:**
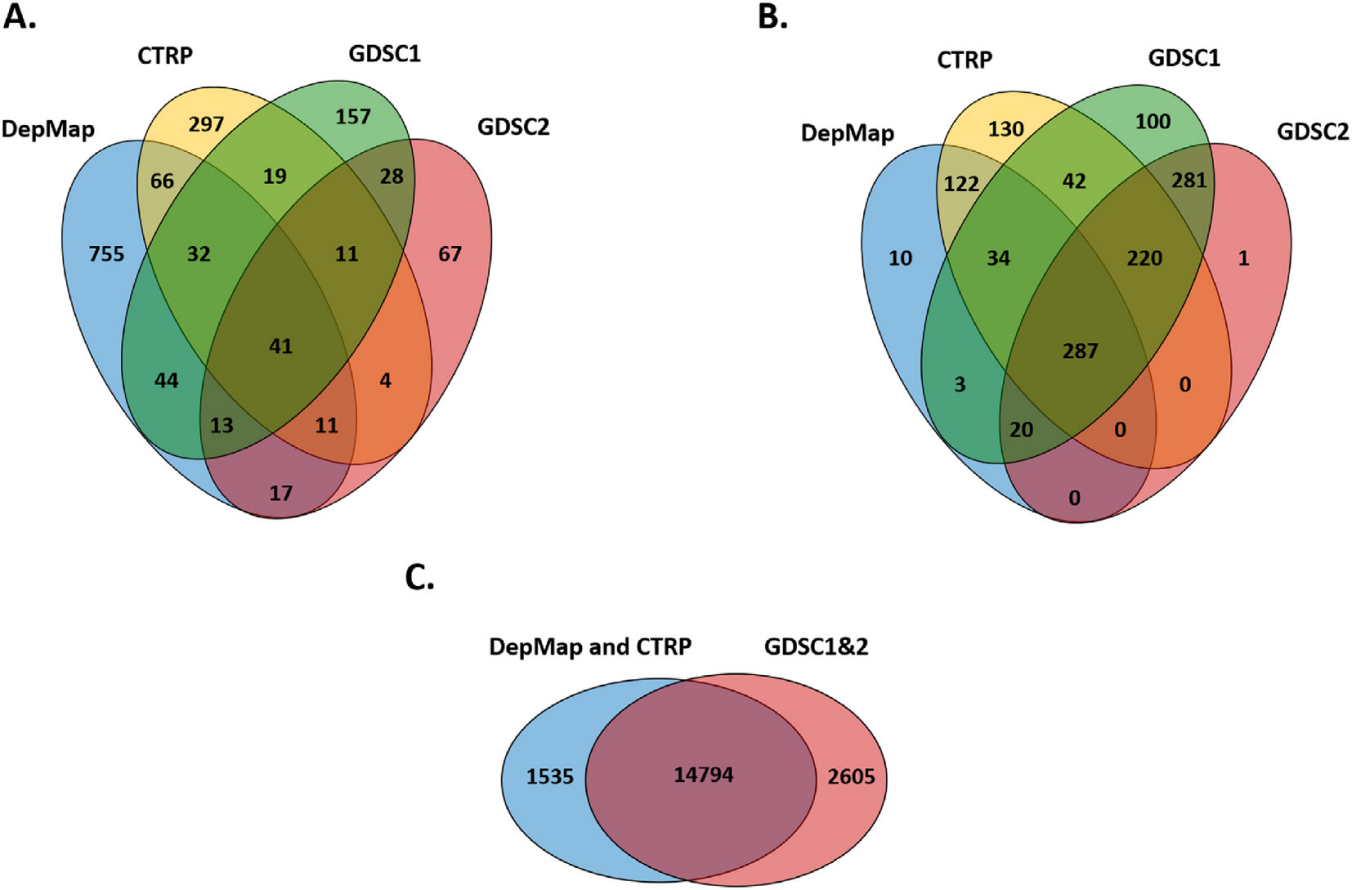
**Venn diagrams comparing the four included datasets,** including The number of investigated agents (A), the number of cell lines (B), and the number of genes (C) used in each included cohort.

The complete aggregated database contains 1562 compounds tested in at least one drug screening projects in a minimum of 100 cell lines with reported IC50 or AUDRC values. Of these, there are 979 compounds in the DepMap project, 481 compounds in the CTRP, 345 compounds in the GDSC1, and 192 compounds in the GDSC2 projects. A total of 286 (18.3%) compounds were tested in at least two drug screening projects and 41 (2.6%) agents were tested in all four projects (Fig. 2A).

The database contains a total of 120 FDA approved anticancer agents. Half of the authorized therapeutic products (n = 61) were categorized as a chemotherapy with antimetabolites being the most common (n = 16). A second major group in medicines authorized for oncology are the targeted therapies (n = 52) and the vast majority (n = 47) of these is involved in the inhibition of a signaling pathway. A complete list of all available oncology licensed compounds is presented in Table 1.

**Table 1:** List of all FDA approved oncology drugs with available *in vitro* resistance data.

Agent	Mechanism of action	Target/Classification	Category
5-fluorouracil	antimetabolite	DNA	chemotherapy
abemaciclib	CDK inhibitor	CDK inhibitor	targeted
abiraterone	antiandrogen	hormonal	hormonal
afatinib	EGFR inhibitor	signal transduction inhibitor	targeted
alectinib	ALK inhibitor	signal transduction inhibitor	targeted
alpelisib	PI3K inhibitor	signal transduction inhibitor	targeted
axitinib	anti-angiogenesis	signal transduction inhibitor	targeted
azacitidine	antimetabolite, hypomethylating agent	DNA	chemotherapy
belinostat	HDAC inhibitor	HDAC inhibitor	chemotherapy
bendamustine	alkylating agent	DNA	chemotherapy
bexarotene	retinoid receptor agonist	differentiating agent	miscellaneous
bicalutamide	antiandrogen	hormonal	hormonal
binimetinib	MEK inhibitor	signal transduction inhibitor	targeted
bleomycin	antitumor antibiotic	DNA	chemotherapy
bortezomib	proteasome inhibitor	proteasome inhibitor	chemotherapy
bosutinib	BCR-ABL inhibitor	signal transduction inhibitor	targeted
brigatinib	ALK inhibitor	signal transduction inhibitor	targeted
busulfan	alkylating agent	DNA	chemotherapy
cabazitaxel	antimicrotubular agent	DNA	chemotherapy
cabozantinib	multiple receptor tyrosine kinase inhibitor	signal transduction inhibitor	targeted
carfilzomib	proteasome inhibitor	proteasome inhibitor	chemotherapy
carmustine	alkylating agent	DNA	chemotherapy
cetuximab	EGFR inhibitor	signal transduction inhibitor	targeted
chlorambucil	alkylating agent	DNA	chemotherapy
cisplatin	platinum analog	DNA	chemotherapy
cladribine	antimetabolite	DNA	chemotherapy
clofarabine	antimetabolite	DNA	chemotherapy
cobimetinib	MEK inhibitor	signal transduction inhibitor	targeted
crizotinib	multiple receptor tyrosine kinase inhibitor	signal transduction inhibitor	targeted
cyclophosphamide	alkylating agent	DNA	chemotherapy
cytarabine	antimetabolite	DNA	chemotherapy
dabrafenib	BRAF inhibitor	signal transduction inhibitor	targeted
dacarbazine	alkylating agent	DNA	chemotherapy
dacomitinib	EGFR inhibitor	signal transduction inhibitor	targeted
dactinomycin	antitumor antibiotic	DNA	chemotherapy
dasatinib	BCR-ABL inhibitor	signal transduction inhibitor	targeted
daunorubicin	antitumor antibiotic	DNA	chemotherapy
decitabine	antimetabolite	DNA	chemotherapy
docetaxel	antimicrotubular agent	DNA	chemotherapy
doxorubicin	antitumor antibiotic	DNA	chemotherapy
epirubicin	antitumor antibiotic	DNA	chemotherapy
erdafitinib	FGFR inhibitor	signal transduction inhibitor	targeted
erlotinib	EGFR inhibitor	signal transduction inhibitor	targeted
estramustine	antimicrotubular agent	DNA	chemotherapy
etoposide	topoisomerase inhibitor	DNA	chemotherapy
etoposide-phosphate	topoisomerase inhibitor	DNA	chemotherapy
everolimus	mTOR inhibitor	signal transduction inhibitor	targeted
fedratinib	JAK inhibitor	signal transduction inhibitor	chemotherapy
floxuridine	antimetabolite	DNA	chemotherapy
fludarabine	antimetabolite	DNA	chemotherapy
fulvestrant	antiestrogen	hormonal	hormonal
gefitinib	EGFR inhibitor	signal transduction inhibitor	targeted
gemcitabine	antimetabolite	DNA	chemotherapy
hydroxyurea	antimetabolite	DNA	chemotherapy
ibrutinib	BTK inhibitor	signal transduction inhibitor	targeted
idarubicin	antitumor antibiotic	DNA	chemotherapy
idelalisib	PI3K inhibitor	signal transduction inhibitor	targeted
ifosfamide	alkylating agent	DNA	chemotherapy
imatinib	BCR-ABL inhibitor	signal transduction inhibitor	targeted
Irinotecan	topoisomerase inhibitor	DNA	chemotherapy
ixabepilone	antimicrotubular agent	DNA	chemotherapy
ixazomib	proteasome inhibitor	proteasome inhibitor	chemotherapy
lapatinib	ERBB inhibitor	signal transduction inhibitor	targeted
lenalidomide	immunomodulatory	miscellaneous	miscellaneous
lenvatinib	multiple receptor tyrosine kinase inhibitor	signal transduction inhibitor	targeted
mechlorethamine	alkylating agent	DNA	chemotherapy
melphalan	alkylating agent	DNA	chemotherapy
mercaptopurine	antimetabolite	DNA	chemotherapy
methotrexate	antimetabolite	DNA	chemotherapy
midostaurin	FLT3 inhibitor	signal transduction inhibitor	targeted
mitomycin-c	antitumor antibiotic	DNA	chemotherapy
mitoxantrone	antitumor antibiotic	DNA	chemotherapy
nelarabine	antimetabolite	DNA	chemotherapy
neratinib	ERBB inhibitor	signal transduction inhibitor	targeted
nilotinib	BCR-ABL inhibitor	signal transduction inhibitor	targeted
niraparib	PARP inhibitor	signal transduction inhibitor	targeted
olaparib	PARP inhibitor	signal transduction inhibitor	targeted
mepesuccinate	BCR-ABL inhibitor	signal transduction inhibitor	targeted
osimertinib	EGFR inhibitor	signal transduction inhibitor	targeted
oxaliplatin	platinum analog	DNA	chemotherapy
paclitaxel	antimicrotubular agent	DNA	chemotherapy
palbociclib	CDK inhibitor	CDK inhibitor	targeted
panobinostat	HDAC inhibitor	HDAC inhibitor	chemotherapy
pazopanib	multiple receptor tyrosine kinase inhibitor	signal transduction inhibitor	targeted
pemetrexed	antimetabolite	DNA	chemotherapy
ponatinib	BCR-ABL inhibitor	signal transduction inhibitor	targeted
pralatrexate	antimetabolite	DNA	chemotherapy
procarbazine	alkylating agent	DNA	chemotherapy
regorafenib	multiple receptor tyrosine kinase inhibitor	signal transduction inhibitor	targeted
ribociclib	CDK inhibitor	CDK inhibitor	targeted
romidepsin	HDAC inhibitor	HDAC inhibitor	chemotherapy
rucaparib	PARP inhibitor	signal transduction inhibitor	targeted
selinexor	XPO inhibitor	nuclear export inhibitor	targeted
selumetinib	MEK inhibitor	signal transduction inhibitor	targeted
sirolimus	mTOR inhibitor	signal transduction inhibitor	targeted
sonidegib	hedgehog inhibitor	signal transduction inhibitor	targeted
sorafenib	multiple receptor tyrosine kinase inhibitor	signal transduction inhibitor	targeted
sunitinib	multiple receptor tyrosine kinase inhibitor	signal transduction inhibitor	targeted
talazoparib	PARP inhibitor	signal transduction inhibitor	targeted
tamoxifen	antiestrogen	hormonal	hormonal
tazemetostat	histone lysine methyltransferase inhibitor	methyltransferase inhibitor	targeted
temozolomide	alkylating agent	DNA	chemotherapy
temsirolimus	mTOR inhibitor	signal transduction inhibitor	targeted
teniposide	topoisomerase inhibitor	DNA	chemotherapy
thioguanine	antimetabolite	DNA	chemotherapy
tipiracil	antimetabolite	DNA	chemotherapy
tirbanibulin	microtubule inhibitor	DNA	chemotherapy
tivozanib	anti angiogenesis	signal transduction inhibitor	targeted
topotecan	topoisomerase inhibitor	DNA	chemotherapy
toremifene	antiestrogen	hormonal	hormonal
trametinib	MEK inhibitor	signal transduction inhibitor	targeted
tucatinib	ERBB inhibitor	signal transduction inhibitor	targeted
valrubicin	topoisomerase inhibitor	DNA	chemotherapy
vandetanib	multiple receptor tyrosine kinase inhibitor	signal transduction inhibitor	targeted
venetoclax	BCL2 inhibitor	signal transduction inhibitor	targeted
vinblastine	microtubule inhibitor	DNA	chemotherapy
vincristine	microtubule inhibitor	DNA	chemotherapy
vinorelbine	microtubule inhibitor	DNA	chemotherapy
vismodegib	hedgehog inhibitor	signal transduction inhibitor	targeted
vorinostat	HDAC inhibitor	HDAC inhibitor	chemotherapy

The database also includes therapeutic agents that are licensed for non-oncological indications (n = 233) as well as compounds that are in the experimental and investigational phase (n = 1209).

### 3.2 Cell lines in the database

Regarding the cell lines in the database, a total of 1250 cell lines were utilized in at least one source dataset. Of these, there are 835 cell lines in the CTRP, 476 in the DepMap, 987 in the GDSC1, and 809 in the GDSC2 projects. A total of 1009 (80.7%) cell lines were tested in at least two drug screening projects and 287 (22.9%) cell lines are available in each source dataset (Fig. 2B). In order to have an adequate sample size for the analyses, certain tumor subtypes were grouped together to create a total of 32 subgroups. A complete list of all 1250 cell lines available in the platform is presented in Supplemental Table S1.

### 3.3 Gene expression database

The gene expression data table of the CCLE cell lines used by the CTRP and DepMap projects contains 19,148 unique HGNC identifiers, while the gene expression data table used for the GDSC1 and GDSC2 projects contains 17,399 unique HGNC identifiers. Genes whose expression was zero in more than half of the tested cell lines were excluded (n = 2819) from the gene expression table of the CCLE cell lines resulting in a total of 16,329 genes with expression values in the database (Fig. 2C).

### 3.4 Ranking of genes associated with ERBB2 inhibition resistance

Two drugs in the DrugBank database targeting the ERBB2 tyrosine kinase domain with a known IC50 or AUDRC values were evaluated in all four included datasets, afatinib and lapatinib. The analysis was performed in each included source cohort separately by using the integrated database and platform to uncover gene expression-based markers of resistance in solid tumors.

Of the three ERBB receptors, the expression of the ERBB2 and ERBB3 genes had a significant association with the therapeutic response for both drugs regardless of the basis of categorization (IC50 or AUDRC). The EGFR (ERBB1) gene had no statistically significant associations in two datasets for lapatinib and in one dataset for afatinib treatment. Detailed results can be found in Table 2. When generating the list of cell lines with the highest sensitivity and resistance, we used both IC50 and AUDRC based classifications. The lists of top ten cell lines are presented as Table 3.

**Table 2:** ROC AUC results and Mann-Whitney test p-values of ERBB receptor tyrosine kinase targeting agents using tertile IC50 and AUDRC based categorization of therapeutic response in each dataset separately.

Response based on	Dataset	EGFR		ERBB2		ERBB3	
		Afatinib	Lapatinib	Afatinib	Lapatinib	Afatinib	Lapatinib
lower vs upper tertile of IC50	DEPMAP	0.659 (3.7e-06)	0.616 (1.8e-03)	0.735 (8.6e-12)	0.787 (1.3e-14)	0.672 (5.7e-07)	0.679 (1.1e-07)
	GDSC1	0.639 (8.0e-10)	0.741 (2.5e-08)	0.658 (2.4e-12)	0.770 (4.1e-10)	0.587 (1.1e-04)	0.609 (5.5e-03)
	GDSC2	n.s.	n.s.	0.619 (2.1e-05)	0.577 (7.4e-03)	0.564 (2.3e-02)	n.s.
	CTRP	not applicable	not applicable	not applicable	not applicable	not applicable	not applicable
lower vs upper tertile of AUDRC	DEPMAP	0.679 (2.0e-07)	0.655 (3.2e-05)	0.761 (3.8e-14)	0.797 (2.0e-15)	0.652 (9.7e-06)	0.694 (1.9e-07)
	GDSC1	0.716 (1.6e-16)	0.728 (3.9e-06)	0.774 (1.1e-25)	0.784 (9.2e-09)	0.683 (2.3e-12)	0.628 (9.6e-03)
	GDSC2	0.592 (8.2e-04)	0.574 (8.5e-03)	0.657 (1.4e-08)	0.617 (3.2e-05)	0.595 (6.2e-04)	0.575 (8.1e-03)
	CTRP	0.681 (1.9e-10)	0.614 (7.1e-05)	0.715 (3.8e-14)	0.670 (2.8e-09)	0.659 (2.0e-08)	0.694 (1.2e-11)

n.s.: not significant.

**Table 3:** **TOP10 lapatinib treated sensitive** (upper panel) **and resistant** (lower panel) **cell lines from the CTRP database.**

Cell line	Disease	Standardized AUDRC
NCIN87	Gastric Cancer/Adenocarcinoma	0.178
HCC2218	Breast Cancer/Breast Ductal Carcinoma	0.185
LC1F	Non-Small Cell Lung Cancer (NSCLC)	0.213
ZR7530	Breast Cancer/Breast Ductal Carcinoma	0.222
SNU175	Colon adenocarcinoma	0.228
YD10B	Head and Neck Cancer/Squamous Cell Carcinoma	0.236
HCC2935	Non-Small Cell Lung Cancer (NSCLC)	0.251
UBLC1	Bladder carcinoma	0.251
TE617T	Rhabdomyosarcoma	0.255
NUGC4	Gastric adenocarcinoma	0.257

Cell line	Disease	Standardized AUDRC
CAL120	Breast carcinoma	0.586
MHH-CALL-4	Acute Lymphoblastic Leukemia (ALL); B-cell	0.582
BFTC-909	Renal Carcinoma; transitional cell	0.576
KMM1	Multiple myeloma	0.547
BEN	Non-Small Cell Lung Cancer (NSCLC)	0.547
HEC265	Endometrial adenocarcinoma	0.546
KARPAS620	Multiple myeloma	0.545
RERFLCAD1	Non-Small Cell Lung Cancer (NSCLC)	0.543
DLD1	Colon adenocarcinoma	0.542
TOV21G	Clear cell adenocarcinoma of the ovary	0.538

**Fig. 3:**
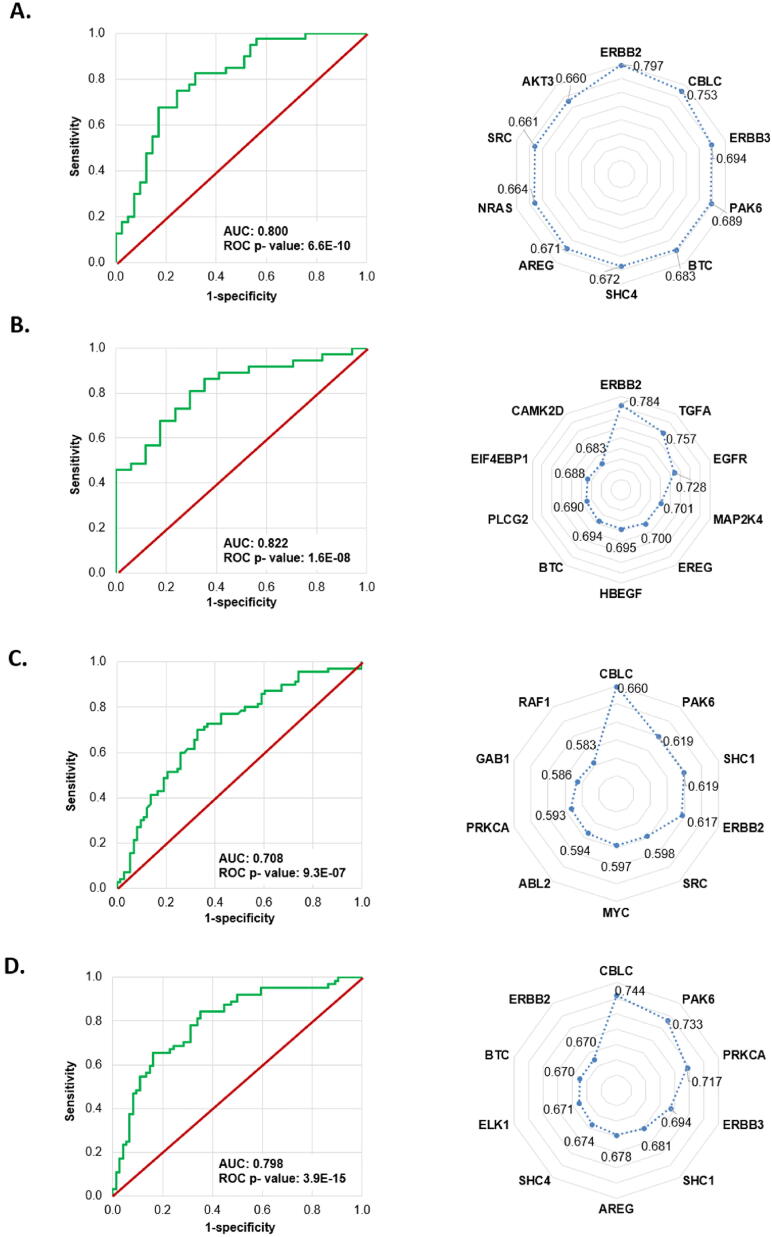
**ROC curves of the random forest models in the test sets and radar chart of the most significant genes correlated with lapatinib resistance in each dataset,** including DepMap (A), GDSC1 (B), GDSC2 (C), and CTRP (D). The values presented in the radar chart are the ROC AUC values for the individual genes.

**Fig. 4:**
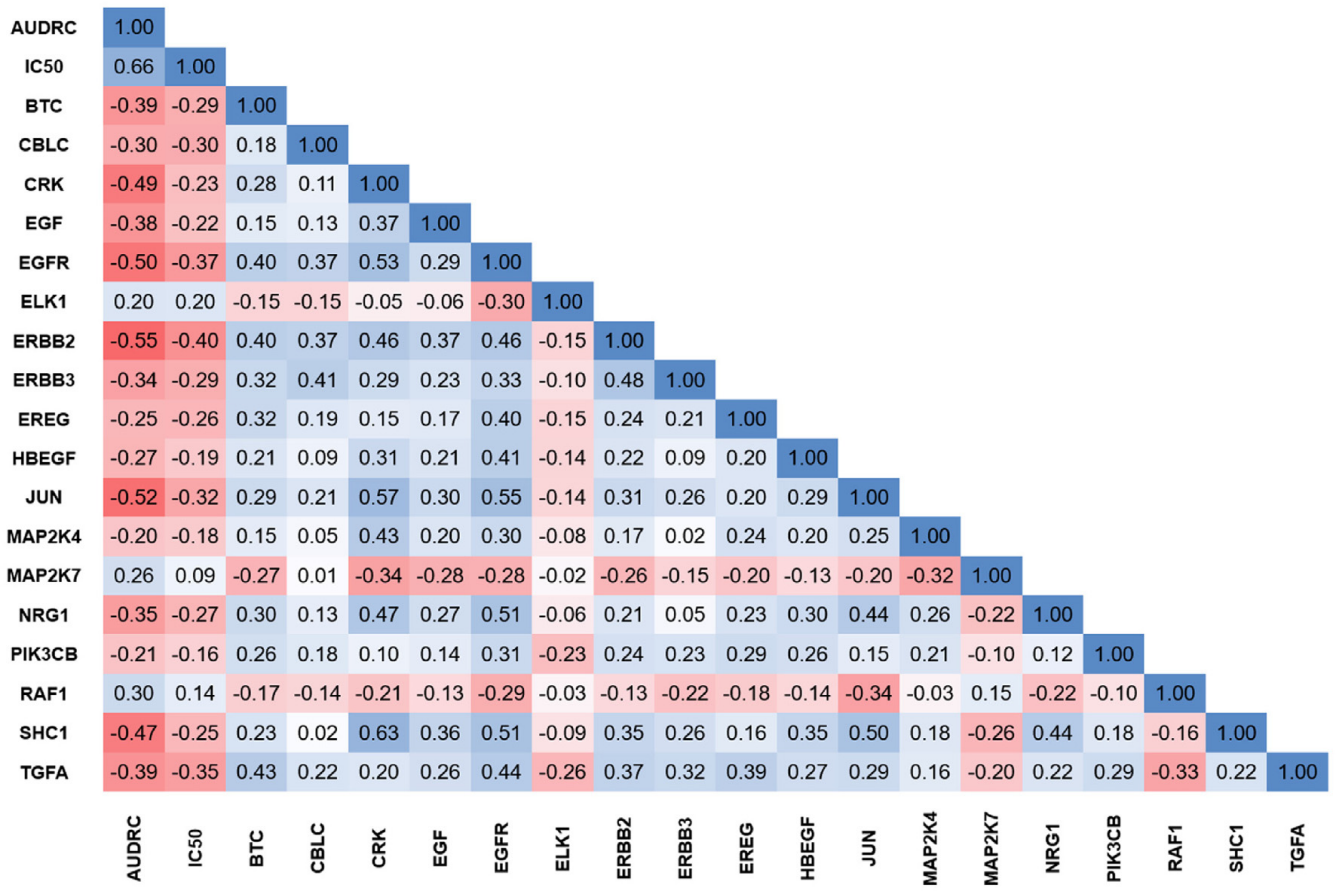
**Correlation between genes related to resistance against the ERBB tyrosine kinase inhibitor lapatinib.** A correlation matrix between drug screening results (AUDRC and IC50) and gene expressions using the GDSC1 dataset is shown. The chart includes only significant genes of the KEGG ERBB pathway for which the Spearman correlation coefficient (when compared to AUDRC) was ≤ -0.20 or ≥ 0.20.

From the 87 genes included in the ERBB pathway we found 25, 30, 24, and 40 genes with significant association with response to lapatinib treatment in DepMap, GDSC1, GDSC2, and CTRP datasets, respectively. The best performing model was observed in the GDSC1 dataset with an overall accuracy of 0.778 and a ROC AUC of 0.822 (p = 1.6E-08). Summary table and radar charts based on the ROC AUC values as well as the ROC plots of the combined models are presented in Fig. 3 and Table 4. Of the significant variables, ERBB2 in the DEPMAP (r = −0.44, p = 7.27E-13) and GDSC1 (r = −0.55, p = 9.99E-22) data set, and CBLC in the GDSC2 (r = −0.27, p = 1.22E-09) and CTRP data sets (r = −0.37, p = 6.89E-18) showed the strongest correlation with the AUDRC values. A correlation matrix between drug screening results (AUDRC and IC50) and gene expression can reveal the influence of individual genes on each other. In Fig. 4 we show a chart depicting only significant genes for which the Spearman correlation coefficient (when compared to AUDRC) was below −0.20 or over ≥ 0.20 in the GDSC1 dataset.

**Table 4:** Summary performance of random forest models for lapatinib resistance in the test set in each dataset.

Dataset	Number of cell lines	Accuracy	Kappa	Sensitivity	Specificity	Precision	ROC AUC	ROC AUC p-value
DepMap	240	0.741	0.482	0.683	0.800	0.778	0.800	6.60E-10
GDSC1	160	0.778	0.450	0.529	0.892	0.692	0.822	1.60E-08
GDSC2	422	0.671	0.344	0.630	0.714	0.697	0.708	9.30E-07
CTRP	409	0.710	0.417	0.730	0.688	0.730	0.798	3.90E-15

## 4 Discussion

Resistance against systemic therapy is a main limitation of current cancer treatment. The utilization of *in vitro* models can provide two important advantages: one can explore the off-target effects of non-oncology drugs related to their potential anticancer repurposing [20] and one can pinpoint new biomarkers of resistance to established agents. Drug repurposing is a common concept also reflected by the numerous studies included in the oncology drug repurposing database [21].

The aim of our study was to enable straightforward utilization of *in vitro* results by establishing a tool to link gene expression and drug sensitivity in a cohort of cell lines from four large cohorts. The complete analysis platform is set up in a way that all available databases and all available cell lines will be used regardless of the selected drug. In addition to the analysis of single genes, we also established a pipeline for the ranking and validation of gene signatures. To enable prompt utilization of the platform we extended our online predictive biomarker discovery application, which previously used clinical samples from breast [18], ovarian [22], glioblastoma [23] and colorectal cancer patients to link gene expression and therapeutic response.

To assess the role of different pathways and cancer hallmark genes in therapeutic responses of drugs we have set up a machine learning-based ranking and validation and utilized this feature to evaluate genes related to anti-ERBB2 therapy resistance. In this, genes related to the resistance against two tyrosine kinase inhibitors were investigated, afatinib and lapatinib. Afatinib is an orally administered irreversible inhibitor of ERBB1 (EGFR), ERBB2, and ERBB4 first approved in 2013 [24]. The ERBB1 and ERBB2 inhibitor lapatinib was approved in 2007 after it showed improved outcome in breast cancer patients whose tumors expressed the ERBB2 (HER2) receptor [25]. Although all four ERBB receptors were implicated in cancer, only ERBB1, ERBB2, and ERBB4 have intracellular tyrosine kinase domains [26]. Despite high success rate, a significant proportion of patients develop resistance against these tyrosine kinase inhibitors [27].

Here, by analyzing *in vitro* data, we pinpoint the genes with the highest correlation to resistance against lapatinib and afatinib. In particular, the strongest genes were ERBB2 itself and Cbl Proto-Oncogene C (CBLC). Cbl proteins ubiquitinate and downregulate other tyrosine kinases and regulate ERBB signal transduction [28]. The expression of CBLC is higher in different tumor types including lung, pancreatic, breast, and colorectal cancer cells and has been suggested as a therapeutic target in lung adenocarcinoma [29]. Our results are strongly supported by a previously described link between CBLC and resistance against lapatinib [30]. A third gene among the most significant hits across multiple datasets was PAK6, a gene encoding a serine/threonine-protein kinase. A previous study utilizing HER2 positive cell lines identified the Akt-signaling pathway in cell lines resistant against ERBB2 inhibitors and suggested PAK6 as a biomarker of resistance [31]. Without further elaboration on individual gens we have to emphasize the high proportion of overlapping hits among the different analyses. These results suggest that the resistance mechanisms converge on a few genes and thus provide a support for the utilization of predictive biomarkers for anti-ERBB tyrosine kinase domain inhibitor therapy.

Another important observation is the superior performance of the random-forest derived single signature when compared to individual genes. The signature had higher AUC value than any gene with the exception of ERBB2 itself in the DepMap cohort. The online analysis portal enables the setup of such resistance-associated signatures for each available drug in an automated manner – see, for example, a previous signature of resistance against EGFR inhibitors manually identified in lung cancer [32].

Notable, some analysis for the investigated datasets are already available at the original repositories. In addition, some previous tools enable the analysis of multiple cell line cohorts as well. The GEMiCCL – Gene Expression and Mutations in Cancer Cell Lines portal was set up to mine and visualize gene expression and mutation data of cell lines [33]. The CellminerCDB is a pharmacogenomic data portal primarily based on the NCI-60 cell lines which integrates multiple layers of data [34]. The web portal we present here has a novel unique place among these resources because we have incorporated more recent datasets, we provide a straightforward automated selection for the investigated genes and we also provide a machine learning algorithm for the data analysis.

There are a few limitations to our study. Firstly, only the reported IC50 and AUDRC valued were used to determine the sensitivity or resistance of a cell line to a particular therapy. Using a fixed cutoff of tertiles for determining sensitivity/resistance might be looked upon as artificial. A second limitation is that not all agents are measured in each cell line, thus, depending on the applied filtering, some drugs cannot be investigated by the proposed pipeline. A third limitation is the utilization of transcriptomic data only – some of the results may be affected by mutations in various genes in individual cell lines. For example, we chose afatinib and lapatinib for our analyses. Afatinib was approved for metastatic NSCLC tumors with L858R variants or exon 19 deletion. The cross-tumor analysis of the data for this inhibitor presented in Table 2 could be affected by the proportion of the cell lines carrying EGFR variants affecting sensitivity to afatinib and by the proportion of NSCLC cell lines in each dataset. Multiple ERBB2 mutations may also affect sensitivity or resistance to lapatinib.

Overall, we have collected and created a unified analysis interface enabling simultaneous mining of four cancer cell line database. The registration-free web application is available at http://www.rocplot.com/cells. We utilized this platform to rank genes correlated to resistance against ERBB2 tyrosine kinase domain inhibitors.

## CRediT authorship contribution statement

**János Tibor Fekete:** Conceptualization, Data curation, Formal analysis, Investigation, Methodology, Resources, Software, Supervision, Validation, Visualization, Writing – original draft, Writing – review & editing. **Balázs Győrffy:** Conceptualization, Formal analysis, Funding acquisition, Methodology, Project administration, Resources, Software, Supervision, Validation, Visualization, Writing – original draft, Writing – review & editing.

## Declaration of Competing Interest

The authors declare that they have no known competing financial interests or personal relationships that could have appeared to influence the work reported in this paper.
